# Characterization of durum wheat resistance against leaf rust under climate change conditions of increasing temperature and [CO_2_]

**DOI:** 10.1038/s41598-023-49118-w

**Published:** 2023-12-12

**Authors:** Rafael Porras, Cristina Miguel-Rojas, Ignacio J. Lorite, Alejandro Pérez-de-Luque, Josefina C. Sillero

**Affiliations:** 1 Area of Plant Breeding and Biotechnology, IFAPA Alameda del Obispo, Avda. Menéndez Pidal S/N, 14004 Córdoba, Spain; 2 Area of Natural and Forest Resources, IFAPA Alameda del Obispo, Avda. Menéndez Pidal S/N, 14004 Córdoba, Spain

**Keywords:** Biotic, Heat

## Abstract

Durum wheat cultivation in Mediterranean regions is threatened by abiotic factors, mainly related to the effects of climate change, and biotic factors such as the leaf rust disease. This situation requires an in-depth knowledge of how predicted elevated temperatures and [CO_2_] will affect durum wheat-leaf rust interactions. Therefore, we have characterised the response of one susceptible and two resistant durum wheat accessions against leaf rust under different environments in greenhouse assays, simulating the predicted conditions of elevated temperature and [CO_2_] in the far future period of 2070–2099 for the wheat growing region of Cordoba, Spain. Interestingly, high temperature alone or in combination with high [CO_2_] did not alter the external appearance of the rust lesions. However, through macro and microscopic evaluation, we found some host physiological and molecular responses to infection that would quantitatively reduce not only pustule formation and subsequent infection cycles of this pathogen, but also the host photosynthetic area under these predicted weather conditions, mainly expressed in the susceptible accession. Moreover, our results suggest that durum wheat responses to infection are mainly driven by temperature, being considered the most hampering abiotic stress. In contrast, leaf rust infection was greatly reduced when these weather conditions were also conducted during the inoculation process, resembling the effects of possible heat waves not only in disease development, but also in fungal germination and penetration success. Considering this lack of knowledge in plant-pathogen interactions combined with abiotic stresses, the present study is, to the best of our knowledge, the first to include the effects of the expected diurnal variation of maximum temperature and continuous elevated [CO_2_] in the durum wheat-leaf rust pathosystem.

## Introduction

Wheat is considered one of the most important crops in the world, accounting for 771 million tons of production on 221 million hectares in 2021 and represents an essential source of calories and protein in the human diets^1^. Therefore, the predicted expansion of the global population up to 9.6 billion people by 2050^2^ would imply an increase of the global demand of wheat consumption. However, accomplishing this objective of production is a challenging task because current wheat yields are limited by diverse abiotic and biotic constraints.

Abiotic stresses, such as drought or high temperatures, affect wheat cultivation depending on the region and environmental conditions, causing physiological and biochemical alterations that ultimately reduce wheat yields, even more than biotic stresses^3,4^. Indeed, environmental conditions have suffered variations due to global climate change^5^, which could be considered an important constraint for plant growth and development through abiotic stresses such as high temperature and drought^6^, reducing wheat yield by 20–30% due to the impact of extreme events^4^. Concretely, the increase in carbon dioxide concentration [CO_2_] is expected to increase global temperature by up to 3.7 °C^5^, and, according to projections for Europe, this increase could be between 4.5 and 5.5 °C, depending on the CO_2_ emission scenario^7^. In addition, wheat is constantly exposed to biotic stresses, which are a major constraint to wheat production worldwide^8–10^. Amongst them, plant diseases cause more than 21% of wheat losses on average^9^, with fungal pathogens such as wheat rusts considered to be the most detrimental^11–13^.

Leaf (brown) rust, caused by the biotrophic fungus *Puccinia triticina* Erikss., is one of the most common and harmful rust of wheat^14,15^. Leaf rust infections reduce the photosynthetic activity of infected leaves, which usually results in yield losses (~ 8.6 million tons annually^14^) through decreasing number of kernels and lower kernel weights^16^. Currently, the most efficient, sustainable, and nonchemical approach to face leaf rust is through genetic breeding^17^. In fact, several genes of resistance to *P. triticina* have been isolated, identified and named as *Lr* genes (~ 80)^18^. Some genes are race-specific, acting in a gene-for-gene manner with the pathogen, and are associated with a qualitative hypersensitive response (HR), while others are non-race specific and develop a partial resistance response (PR), showing durable incomplete resistance, characterised by a slower development of the disease^19^. Thus, to discover potential sources of resistance, the identification of both macro and microscopic components of resistance is considered essential. In fact, although leaf rust evaluation has been generally achieved through visual assessment^19,20^, image analysis has emerged as a precise tool for quantitatively evaluating wheat rusts^21–23^. Additionally, histopathological methods allow the identification of microscopic components of resistance to wheat rusts such as generation of reactive oxygen species (ROSs), or the presence and development of a hypersensitive response (HR) amongst others^20,24,25^. In summary, both macro and microscopic evaluations are necessary not only in finding new sources of resistance, but also in the detailed evaluation of effects of abiotic factors in wheat-leaf rust interactions under future climate conditions.

Indeed, wheat-pathogen interactions are likely to be influenced by future climate change alterations in temperature, [CO_2_] and water regimes^17^, which would modify plant development and resistance pathways, on one side, and pathogen virulence mechanisms and life cycle, on the other side^26^. Thus, disease risk simulation studies postulate as an essential tool to predict these climate impacts on wheat-pathogen interaction worldwide^27–29^, but they involve a certain degree of uncertainty^30^. This, combined with a lack of realistic field studies about the effects resulting from the combination of simultaneous abiotic and biotic stresses in wheat^31,32^, make disease predictions under future climate change a challenging task. In fact, these studies are far from finding a similar response about the effects of expected abiotic factors, such as high temperature and elevated [CO_2_], on wheat-pathogen interactions^33–39^, possibly due to diverse plant genotypes, pathogen lifestyles, and/or timing and intensity of simultaneous abiotic factors^32^. In this sense, elevated temperature modulates plant resistance against pathogens, increasing or decreasing it in terms of both basal and race-specific resistance^40^. In the case of wheat, it is known that some resistance genes against the most important rusts (leaf rust, yellow rust and stem rust) are influenced by temperature, being effective only at warm environmental conditions^41–43^. As a consequence, the deployment of this kind of genes requires experiments with specific conditions of increasing temperature in wheat-rust interactions for their identification^44–46^, which would become complex in future breeding studies if weather conditions become warmer.

In addition, plants have a tailored physiological and molecular response to multiple stresses, which cannot be elucidated from studies of individual stresses, mainly due to the occurrence of antagonistic signalling pathways between abiotic and biotic stresses^32,47^, such as the role of some phytohormones in this kind of responses^48^. Eventually, one possible effect derived from the exposure to multiple stresses is that plants that face a prolonged abiotic stress can be more resistant or susceptible to a subsequent pathogen attack. This phenomenon of plant acclimation (or cross-tolerance/susceptibility)^31,48^, shows that plants possess a powerful regulatory system that allows them to adapt quickly to changing environments^49^. In fact, some durum wheat cultivars could prevent or minimise the detrimental effect of higher temperatures through basal or acquired (after acclimation) thermotolerance, synthesising heat shock proteins that improve membrane stability, the use of water and nutrients or assimilate partitioning^50^, which favours at the same time the general health status of plants and the above-mentioned phytohormone cross-tolerance before fungal infection. It is therefore necessary to study empirically how the combined environmental factors expected in the future climate change scenarios would affect plant immunity, pathogen virulence, and disease development in wheat–pathogen interactions.

This is particularly true for several European wheat-growing areas with Mediterranean-like climatic characteristics, such as some Spanish regions, where not only an increase in leaf rust disease is predicted by 2050^29^, but also a major constraint on wheat production due to abiotic factors. Furthermore, as these regions are considered hotspots of climate change, temperature warming, extreme events and changes in precipitation regimes are likely to occur^51,52^. Therefore, this situation would particularly affect the cultivation of durum wheat, which is considered a staple crop in Mediterranean countries^53,54^. In some Spanish growing areas, the appearance of new virulent races of leaf rust^55^ coupled with the threat of climate change^17,26,56^ could severely hamper future durum wheat production.

The aim of this study was to identify macro and microscopic components of resistance against leaf rust in durum wheat accessions with different levels of resistance, using greenhouse experiments that simulated the predicted conditions of increased temperature and [CO_2_] in the far future period of 2070–2099 for the wheat growing region of Cordoba, Spain.

## Methods

### Plant material

In our study we evaluated 45 durum wheat (*T. turgidum* spp. *durum*) accessions against a local isolate, SanEs18/5, of leaf rust (*P. triticina*). The studied accessions were 22 breeding lines, belonging to the wheat breeding program developed at IFAPA (Instituto Andaluz de Investigación y Formación Agraria, Pesquera, Alimentaria y de la Producción Ecológica) Spain, and 23 commercial Spanish cultivars, registered in the Spanish MAPA (Ministerio de Agricultura, Pesca y Alimentación) catalogue (Supplementary Table S1), either recently registered or widely cultivated by Spanish farmers.

### Pathogen isolation

*Puccinia triticina* isolate used in this study was collected in a naturally infected field of the durum wheat variety Sculptur at Santaella (Cordoba, Spain) in 2018. Spores from the infected leaves were inoculated onto uninfected plants of susceptible cultivar Qualidou to purify the inoculum. Plants were placed in a humidity chamber at 21 °C to provide 100% relative humidity (RH) and incubated for 24 h. Then, plants were transferred to a growth chamber at 21 °C day/night with 70% RH and 14-h photoperiod for 9 days. When individual pustules appeared, a single-pustule isolate was obtained and multiplied in 14-day-old Qualidou plants in order to increase the number of spores for further inocula. Plants were inoculated with spores mixed with pure talc (1:20 v/v) using a manual airbrush spray and incubated as described above. Then, spores of leaf rust were collected using a vacuum bomb and stored at − 80 °C until inoculation experiments. Finally, near-isogenic Thatcher lines with known *Lr* genes were inoculated and this leaf rust isolate showed virulence on the following *Lr* genes: *Lr1*, *Lr2c*, *Lr3*, *Lr3bg*, *Lr3ka*, *Lr10*, *Lr11*, *Lr12*, *Lr14a*, *Lr14b*, *Lr18*, *Lr20*, *Lr22a*, *Lr23*, *Lr30*, *Lr33*, *Lr34*, *Lr35*, *Lr37*, *Lr45*, *LrB*; and avirulence on the *Lr* genes: *Lr2a*, *Lr2b*, *Lr9*, *Lr13*, *Lr15*, *Lr16*, *Lr17*, *Lr19*, *Lr21*, *Lr24*, *Lr25*, *Lr26*, *Lr28*, *Lr32*, *Lr36*, *LrW* (Supplementary Table S2).

### Screening of the durum wheat germplasm collection

Seeds of 45 durum wheat accessions were sown in 8 × 7 × 7 cm pots containing a mix (1:1 v/v) of commercial compost (Suliflor SF1 substrate; Suliflor, Radviliškis, Lithuania) and sand. Pots were then placed in trays and incubated in a growth chamber at 21 °C day/night with a 14-h photoperiod for germination. After 12 days, when the second leaf was completely unfolded, four seedlings of each accession were inoculated with leaf rust spores mixed with pure talc (1:20 v/v) using a manual airbrush spray, and incubated in a humidity chamber as described above. A total of 180 plants per experiment (4 biological replicates for each accession) were uniformly inoculated with 80 mg of leaf rust spores. The experiment was performed three times. For disease assessment, the second leaf of each plant was evaluated 9 days post inoculation (dpi). The infection process was recorded as the percentage of each leaf with disease symptoms (pustules, chlorosis and necrosis), referred to as disease severity (DS). In addition, seedling reactions were registered using a disease scoring scale (0–9) for infection type (IT)^57^, where 0 = no visible disease symptoms (immune), 1 = minor chlorotic and necrotic flecks, 2 = chlorotic and necrotic flecks without sporulation, 3–4 = chlorotic and necrotic areas with limited sporulation, 5–6 = chlorotic and necrotic areas with moderate sporulation, 7 = abundant sporulation with moderate chlorosis, 8–9 = abundant and dense sporulation without notable chlorosis and necrosis. Infection types 0–6 were considered resistant, while types 7–9 were considered susceptible.

After this preliminary disease screening, three durum wheat accessions with different responses to infection, ranging from resistant to susceptible, were selected for assessing macro and microscopic components of resistance to leaf rust under baseline (control) and climate change conditions. These accessions were: BL 28, BL 38 and Qualidou.

### Greenhouse conditioning and design of climate environments

Plants of the three selected durum wheat accessions were grown in greenhouses with full environmental control of temperature and [CO_2_], similarly to the study of Porras et al.^58^ for evaluation of Septoria tritici blotch disease. To establish these weather and [CO_2_] conditions, the greenhouses were equipped with air conditioning and dehumidification systems, and CO_2_ supply circuits, all controlled by temperature, humidity, and CO_2_ sensors, with a fully automated CO_2_ injection process to maintain the CO_2_ target levels (Sysclima, version 9.4, INTA CROP TECHNOLOGY S.L., Murcia, Spain). The established weather conditions were designed to resemble a standard spring day, which is the expected growth period of *P. triticina* in the wheat growing area of Cordoba.

Since average temperatures may not always be an accurate predictor of the potential for an infection^26^, in our study we carried out a variation of temperature throughout the day, reaching an established maximum and minimum value. Thus, for the baseline, the maximum and minimum temperature values were obtained from the nearest meteorological station, located in Cordoba and belonging to the Spanish State Meteorological Agency, with average values of 24 °C and 10 °C, respectively. Likewise, the value of [CO_2_] was set at around 420–450 ppm, the level currently observed outdoors. Moreover, in order to define the weather conditions for the far future period (2070–2099), the Representative Concentration Pathway RCP8.5 and an ensemble of five climate models (GFDL-CM3, GISS-E2-R, HadGEM2-ES, MIROC5 and MPI-ESM-MR) were taken into account, resulting in an average maximum and minimum temperatures around 30 °C and 15 °C, respectively, and an average [CO_2_] around 620–650 ppm.

Having established the above weather and [CO_2_] conditions, five sets of plants from three durum wheat accessions were exposed to three different environments, each in separate greenhouses, to assess *P. triticina* infection. Under baseline conditions (environment B), plants were exposed to a maximum temperature of 24 °C and [CO_2_] around 420–450 ppm. For the far future scenario, two possible environments were established: under increasing temperature (environment 1), plants were exposed to a maximum temperature of 30 °C and [CO_2_] around 420–450 ppm; and under increasing temperature and [CO_2_] (environment 2), plants were exposed to a maximum temperature of 30 °C and elevated [CO_2_] around 620–650 ppm.

One set of plants was grown, incubated and maintained for evaluation under baseline weather conditions (set SB), and four sets of plants were grown under far future weather conditions: two sets at elevated temperature and two at both elevated temperature and [CO_2_]. As high temperatures could affect critical phases of the *P. triticina* infection process*,* two of the four sets of plants under far future conditions were inoculated and incubated under baseline weather conditions, before returning to their respective far future conditions (sets S1 and S2, respectively). The other two sets of plants were grown, inoculated, incubated and maintained for evaluation under their corresponding far future weather conditions (sets S1G and S2G, respectively).

### Inoculation assays for evaluation of components of resistance

Seeds of the three selected durum wheat accessions were sown in 30 × 20 × 7 cm trays containing the mix of commercial compost and sand (1:1, v/v) described above. Trays were first incubated at 21 °C with a 14-h photoperiod in a growth chamber to germinate the plants for 6 days, and then, seedlings were transferred to different greenhouse with diverse weather conditions described above (environments B, 1 and 2) for 15 days until the third leaf was completely unfolded. Then, following Sorensen et al.^59^ with minor modifications, third-leaves were fixed horizontally (adaxial surface up) on a foam board with metal clips. A total of 27 leaves (9 per accession) were fixed in each one of the five trays (one per plant set) for subsequent macro and microscopic evaluations, being evaluated a total of 135 leaves per replication. Each tray was inoculated with 4 mg of leaf rust spores (with a spore deposition of 280–300 spores per cm^2^) mixed with pure talc (1:20 v/v) using a settling tower which led to a uniform inoculation of the leaves. Then, trays were covered with black plastic bags to maintain a 100% RH and darkness for the leaf rust inoculation for 24 h. Three sets of plants (SB, S1 and S2) were incubated on environment B, while two sets of plants were incubated on their corresponding environments 1 or 2 (S1G and S2G, respectively). Finally, plastic bags were removed, and plants were kept in their respective environments for 9 days. Macroscopic and microscopic experiments were performed three times each.

### Assessment of macroscopic components of resistance

Leaf segments of 2 cm long from the three selected accessions (Qualidou, BL 28 and BL 38) were marked before the appearance of rust pustules in four leaves per accession, plant set and replication at 5 dpi. Then, the number of visible pustules breaking the leaf epidermis in the marked segments were recorded at different time intervals until the number no longer increased. A portable camera (IPEVO DO-CAM, Sunnyvale, CA, USA) equipped with a hand-lens was used to count the rust pustules, taking photos of leaf segments through different time intervals. Thus, latency period (LP50) was calculated as the number of hours from the day of inoculation to the appearance of 50% of the total pustules breaking leaf epidermis in the marked segments. In addition, five leaves per accession, plant set and replication were detached, placed on black sheets of cardboard, and digitally scanned (Canon CanoScan LiDE 400, Tokyo, Japan) at 1200 ppi of resolution after 9 dpi, similar to Cabrera et al.^21^. The image analysis software Fiji (Wayne Rasband, NIH, MD, USA)^60^ was used for analysing 4 cm^2^ of four leaves per accession. The parameters analysed, based on Porras et al.^23^ for *Puccinia striiformis* with modifications, were Infection Frequency (IF, number of pustules per cm^2^ of leaf), Mean Pustule Size (cm^2^), Total Pustule Area relative to leaf area (%), Total Disease Area (pustule area plus chlorosis and necrosis areas) relative to leaf area (%) and Pustule Development Rate (proportion of pustule area relative to Total Disease Area (%)). Areas of pustules and disease symptoms were determined by the colour thresholding option using the default method with the HSB colour space setting.

### Assessment of microscopic components of resistance

Central leaf segments (~ 6 cm) of third-leaves placed on cork pedestals mentioned above were cut at 5 dpi in four leaves per accession, plant set and replication. Samples were processed as described in Porras et al.^23^ and then examined using a Nikon epifluorescence equipment (Nikon, Tokyo, Japan) with a V-2A filter (excitation filter 380–420 nm, barrier filter 430 nm). Fungal colonies were classified as early-aborted (EA), when spores developed a substomatal vesicle (SSV), a primary infection hypha and no more than 6 haustorial mother cells (HMC), and established (EST), when spores developed a SSV and a primary infection hypha with more than 6 HMC. In both development stages, the presence (+) or absence (−) of plant cell death autofluorescence (necrosis) was considered to establish early aborted colonies associated to necrosis (EA+) or not (EA−), and established colonies associated to necrosis (EST+) or not (EST−). A total of 150 spores in four leaves per accession, plant set and replication were evaluated and classified according to the mentioned fungal stages of development. Only spores which formed an infection site were counted. Fungal stages of development were photographed using a Nikon DS-Fi1 camera (Nikon, Tokyo, Japan). In addition, 40 established infection units in four leaves per accession, plant set and replication were measured in their length (L) and width (W) using a micrometer. In the resistant accession BL 38, only 10–20 established infection units per plant set and replication were measured due to the reduced occurrence of this fungal stage. Colony size (CS) was calculated as the geometric mean of L and W, CS = √(^1^/_4_ × π × L × W)^61^.

### Statistical analysis

The experimental design was developed as randomised blocks. Macroscopic and microscopic parameters whose data did not achieve normality and homogeneity requirements amongst different environments for each accession were transformed for statistical analysis with ANOVA test, and back transformed for presentation. However, parameters whose data could not achieve those requirements using transformations were analysed through nonparametric Kruskal–Wallis test. Thus, data from macroscopic parameter LP50 in accession Qualidou was analysed using ANOVA and Dunnett T3 test, while the rest of macroscopic parameters in this accession were analysed using ANOVA and LSD (Least Significant Difference) tests. Data from IF, Total Pustule Area and Total Disease Area in accession Qualidou were transformed according to the formula y = √(x). Macroscopic data from Total Disease Area were also transformed according to the formula y = log(x) and y = √(x) in BL 28 and BL 38 accessions, respectively, and analysed using ANOVA and LSD test. Finally, macroscopic parameters IF, Mean Pustule Size, Total Pustule Area and Pustule Development Rate in accession BL 28 were analysed using Kruskal–Wallis test. In terms of microscopic parameters, data from percentages of different fungal stages and CS parameters were analysed using ANOVA and Duncan tests for the three selected accessions. Data from microscopic fungal stages EA− and EA+ in accession Qualidou, and EST+ in accession BL 38 were transformed according to the formula y = √(x). Data processing, statistical analyses and figure design were carried out using R software^62^ and Fiji^60^.

## Results

### Response of durum wheat germplasm to leaf rust infection

**Figure 1:**
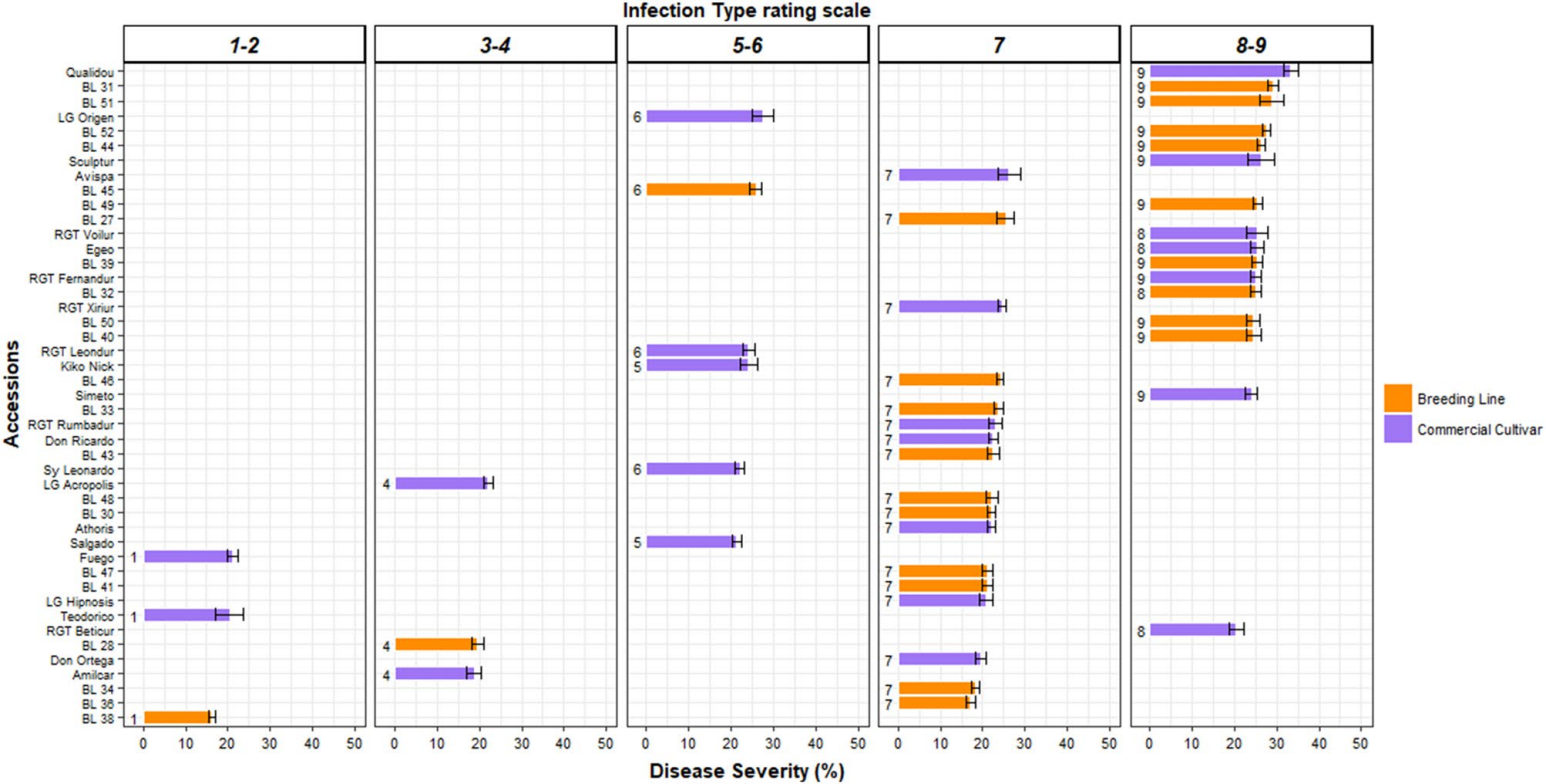
*P. triticina* infection in durum wheat breeding lines and commercial cultivars at 9 dpi. Mean percentage of disease severity (DS), presented in columns, and infection type (IT) rating scale, presented as numbers at the top of the figure. Accessions were arranged according to their mean percentage of DS and classified according to IT in panels. The IT scale is presented according to McNeal et al.^57^, where 0 = no visible disease symptoms (immune), 1 = minor chlorotic and necrotic flecks, 2 = chlorotic and necrotic flecks without sporulation, 3–4 = chlorotic and necrotic areas with limited sporulation, 5–6 = chlorotic and necrotic areas with moderate sporulation, 7 = abundant sporulation with moderate chlorosis, 8–9 = abundant and dense sporulation without notable chlorosis and necrosis. Infection types 0–6 were considered resistant, while types 7–9 were considered susceptible. Error bars represent the standard error calculated from three independent experiments with four replicates each.

The 45 durum wheat accessions were evaluated for disease reactions against *P. triticina* isolate SanEs18/5 and classified according to their percentage of DS and IT (Fig. 1). There was a high proportion of accessions (33 in total) presenting a susceptible response (IT 7–9) under optimum conditions of fungal infection, being 19 of them breeding lines and 14 commercial cultivars, respectively. Amongst them, seventeen accessions showed an IT value of 7, developing abundant sporulation but with the appearance of few chlorosis surrounding the pustules, while the rest of accessions showed IT values of 8 and 9, developing abundant and dense sporulation without chlorosis. Eventually, in the case of breeding lines, almost all (19 out of 22) expressed IT 7–9 values.

The remaining 12 accessions showed diverse values of resistant response to *P. triticina* infection, highlighting accessions with an incompatible response in the form of minor chlorotic flecks (IT 1) such as BL 38 (breeding line), Fuego and Teodorico (commercial cultivars). Other two commercial cultivars (LG Acropolis and Amilcar) and the breeding line BL 28 expressed a high resistant response, showing chlorosis and necrosis surrounding limited sporulation (IT 3–4), whereas other commercial cultivars and the breeding line BL 45 exhibited a moderately resistant response in the form of chlorosis and necrosis surrounding moderate sporulation (IT 5–6).

### Macroscopic components of resistance to *P. triticina* infection under climate change conditions

We selected three suitable accessions for the climate change experiments which present resistant and susceptible reactions against leaf rust. Thus, breeding line BL 38 (IT 1) was chosen as highly resistant, breeding line BL 28 (IT 4) as moderately resistant, and commercial cultivar Qualidou (IT 9) as susceptible. These selected accessions were macroscopically evaluated to characterise components of resistance to leaf rust infection under diverse weather conditions. Thus, we evaluated diverse components of *P. triticina* infection through image analysis, such as IF, Mean Pustule Size, Total Pustule Area, Total Disease Area and Pustule Development Rate (Table 1, Supplementary Fig. S1).

**Table 1:** Macroscopic image analysis of *P. triticina* infection in three selected durum wheat accessions under baseline and climate change environments at 9 dpi.

Accession	Environmental set	IF (Pustules/cm^2^)	Mean pustule size (× 10^–3^ cm^2^)	Total pustule area (%)	Total disease area (%)	Pustule development rate (%)
Qualidou	SB	76.27 (8.67 ± 0.29) c	1.36 ± 0.038 a	10.28 (3.18 ± 0.10) a	20.54 (4.52 ± 0.10) b	49.77 ± 2.08 a
	S1	102.29 (10.09 ± 0.19) a	0.99 ± 0.028 b	10.12 (3.17 ± 0.07) a	28.26 (5.31 ± 0.06) a	35. 80 ± 1.35 b
	S2	89.51 (9.42 ± 0.24) b	0.96 ± 0.026 b	8.63 (2.92 ± 0.08) b	27.31 (5.21 ± 0.10) a	31.51 ± 1.18 b
	S1G	18.98 (4.30 ± 0.19) e	0.97 ± 0.050 b	1.88 (1.34 ± 0.08) c	7.83 (2.77 ± 0.10) d	23.47 ± 1.69 c
	S2G	28.44 (5.28 ± 0.20) d	0.71 ± 0.041 c	2.09 (1.41 ± 0.08) c	12.35 (3.49 ± 0.10) c	17.12 ± 1.70 d
BL 28	SB	1.24 ± 0.28 a	0.25 ± 0.019 a	0.03 ± 0.01 a	6.49 (0.78 ± 0.05) a	0.48 ± 0.10 a
	S1	3.44 ± 1.00 a	0.28 ± 0.019 a	0.10 ± 0.03 a	6.79 (0.80 ± 0.04) a	1.29 ± 0.39 a
	S2	1.00 ± 0.37 ab	0.26 ± 0.015 a	0.03 ± 0.01 ab	5.56 (0.71 ± 0.05) a	0.37 ± 0.14 a
	S1G	0.39 ± 0.13 ab	0.31 ± 0.032 a	0.01 ± 0.00 ab	1.04 (-0.05 ± 0.07) c	1.06 ± 0.38 a
	S2G	0.18 ± 0.08 b	0.25 ± 0.025 a	0.00 ± 0.00 b	2.05 (0.28 ± 0.04) b	0.21 ± 0.09 a
BL 38	SB	–	–	–	1.44 (1.18 ± 0.05) b	–
	S1	–	–	–	2.10 (1.43 ± 0.04) a	–
	S2	–	–	–	2.00 (1.40 ± 0.04) a	–
	S1G	–	–	–	0.47 (0.68 ± 0.03) d	–
	S2G	–	–	–	0.78 (0.87 ± 0.04) c	–

Values are mean ± standard error for five leaves evaluated for each accession and environmental set in three different experiments. Transformed data ± standard error are shown in parenthesis. Data with the same letter within an accession and column are not statistically different (LSD and Kruskal–Wallis tests, *p* < 0.05). Dash (–) means no data were measured since there was no pustule development. SB: plants grown, inoculated, incubated, and maintained for evaluation under baseline weather conditions (24 °C and [CO_2_] around 420–450 ppm). S1 and S2: plants inoculated and incubated under baseline weather conditions, and then maintained for evaluation under far future weather conditions (S1, 30 °C and [CO_2_] around 420–450 ppm; S2, 30 °C and elevated [CO_2_] around 620–650 ppm). S1G and S2G: plants grown, inoculated, incubated, and maintained for evaluation under far future weather conditions (S1G, 30 °C and [CO_2_] around 420–450 ppm; S2G, 30 °C and elevated [CO_2_] around 620–650 ppm).

The susceptible accession Qualidou showed the greatest differences in macroscopic parameters amongst weather conditions. This accession showed the lowest IF value for the S1G (18.98 pustules/cm^2^) set, compared to the SB set (76.27 pustules/cm^2^). In contrast, it developed the highest IF value for the S1 set (102.29 pustules/cm^2^). For IF parameter, all data were statistically different amongst them. For the Mean Pustule Size parameter, this accession developed significantly lower values for all sets considering far future weather conditions than SB set (1.36 × 10^−3^ cm^2^). We observed closer values amongst S1, S1G and S2 sets (around 0.97 × 10^−3^ cm^2^) except for the set S2G (0.71 × 10^−3^  m^2^) which was statistically different. Referred to the Total Pustule Area parameter, accession Qualidou expressed similar values for SB and S1 sets (10.28% and 10.12%, respectively), which were relevantly higher than values in S2 set (8.63%) and, especially, in S1G and S2G sets (1.88% and 2.09%, respectively). In addition, chlorotic and necrotic areas were identified through image analysis, and together with pustule area, were recorded as Total Disease Area (Table 1). Therefore, the highest values in Qualidou accession were expressed in sets S1 and S2 (28.26% and 27.31% values, respectively), being significantly lower in the SB set (20.54%), followed by S2G (12.35%) and S1G (7.83%) sets. Lastly, we determined which fraction of the disease area was considered as pustule area, being this parameter named as Pustule Development Rate. This value was significantly higher for the SB set (49.77%) in comparison to the other sets. The Qualidou accession developed similar values for S1 and S2 sets (35.80% and 31.51%, respectively), whereas S1G and S2G sets showed even more reduced values (23.47% and 17.12%, respectively), both statistically different between them and with the other sets.

BL 28 accession scored reduced values for all macroscopic parameters studied (Table 1). The IF parameter showed the highest value for the S1 set (3.44 pustules/cm^2^), while the lowest one for the S2G set (0.18 pustules/cm^2^). Regarding Mean Pustule Size parameter, we found very similar values amongst sets, ranging from 0.25 × 10^−3^ to 0.31 × 10^−3^ cm^2^. Similarly to IF parameter, Total Pustule Area parameter presented from the highest value for S1 set (0.10%) to the lowest one in S2G set (0.00%). Interestingly, the Total Disease Area values were relevantly higher for all sets in BL 28 accession compared with Total Pustule Area values, in concordance with its supposed moderately resistant behaviour. S1G and S2G sets showed slightly lower values, statistically different between them and with the other sets. Finally, accession BL 28 showed reduced Pustule Development Rate values statistically non-significant.

Leaves from accession BL 38 did not develop pustules in none of the sets of our study (Table 1). Due to this absence of pustules, IF, Mean Pustule Size, Total Pustule Area and Pustule Development Rate parameters were not collected (accounted with dash). Thus, only the Total Disease Area parameter was measured in this accession. The highest values were displayed for S1 and S2 sets (2.10% and 2.00%, respectively), while the lowest ones under S2G and S1G sets (0.78% and 0.47%, respectively).

In addition, we also evaluated the latency period (LP50, Table 2). The susceptible accession Qualidou showed a LP50 value of 172.65 h under environment B (SB set), while plants belonging to S1 and S2 sets expressed shortened LP50 values, with 171.66 h and 167.83 h, respectively. However, S1G and S2G plants, which were inoculated and incubated in environments 1 and 2, showed significantly longer LP50 values than the other three sets (185.67 and 186.21 h, respectively). Unfortunately, accession BL 28 developed such a scarce quantity of pustules with small size in all weather conditions, that an acceptable measurement of LP50 data was not feasible. In this sense, *P. triticina* did not develop pustules either in BL 38 accession, making data collection for LP50 values impossible to carry out, similarly to some parameters of Table 1.

**Table 2:** Latency Period (LP50) in durum wheat accession Qualidou under baseline and climate change environments.

Accession	Environmental set	LP50 (hours)
Qualidou	SB	172.65 ± 1.06 b
	S1	171.66 ± 1.39 bc
	S2	167.83 ± 0.75 c
	S1G	185.67 ± 3.28 a
	S2G	186.21 ± 1.61 a

Values are mean ± standard error for four leaves evaluated for each environmental set in three different experiments. Data with the same letter within a column are not statistically different (Dunnett T3 test, *p* < 0.05). SB: plants grown, inoculated, incubated, and maintained for evaluation under baseline weather conditions (24 °C and [CO_2_] around 420–450 ppm). S1 and S2: plants inoculated and incubated under baseline weather conditions, and then maintained for evaluation under far future weather conditions (S1, 30 °C and [CO_2_] around 420–450 ppm; S2, 30 °C and elevated [CO_2_] around 620–650 ppm). S1G and S2G: plants grown, inoculated, incubated, and maintained for evaluation under far future weather conditions (S1G, 30 °C and [CO_2_] around 420–450 ppm; S2G, 30 °C and elevated [CO_2_] around 620–650 ppm).

### Microscopic components of resistance to *P. triticina* infection under climate change conditions

**Figure 2:**
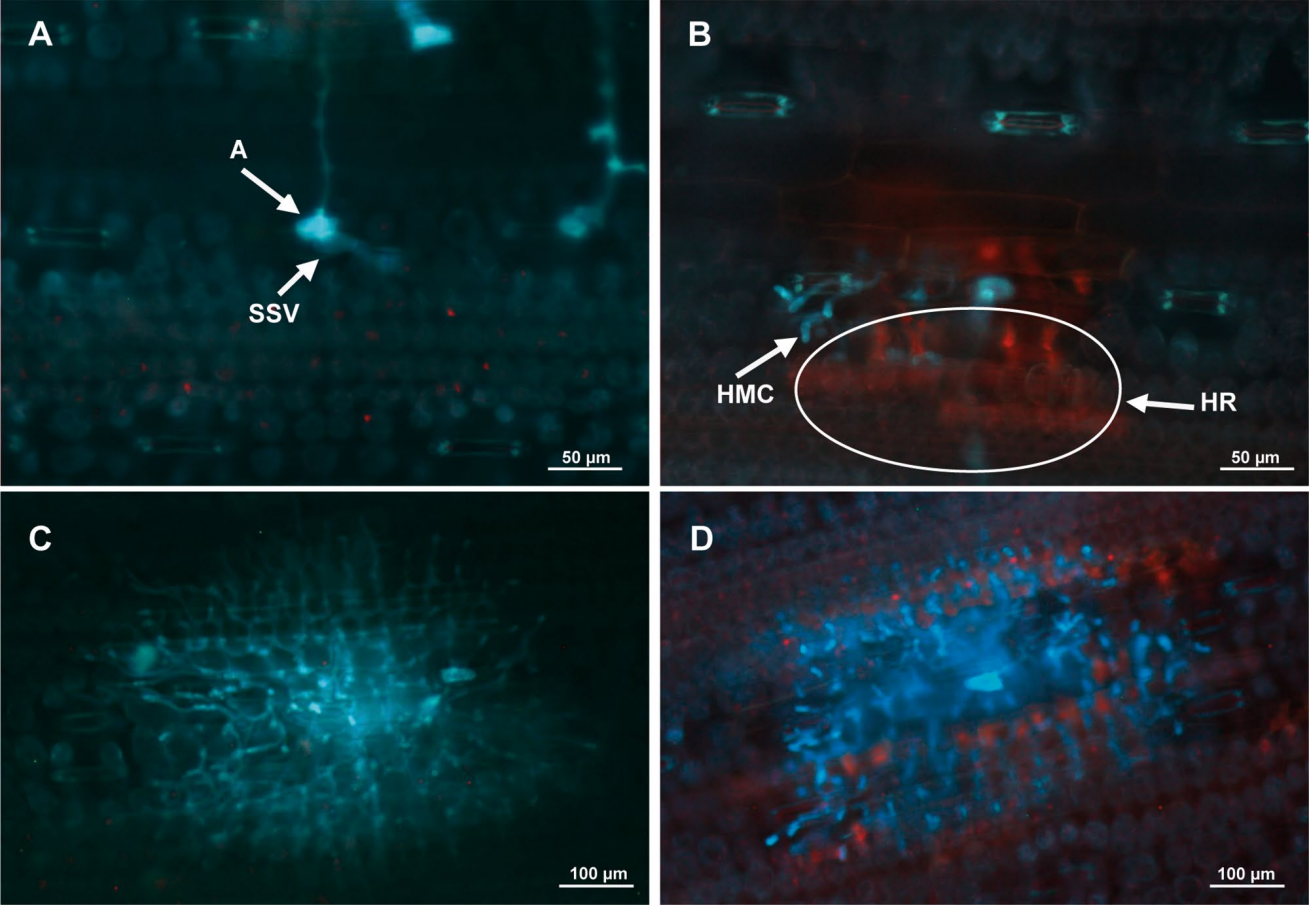
Microscopically observed fungal stages of *P. triticina* and plant cellular responses at 5 dpi were classified as: (**A**) early-aborted colony without necrosis (EA−); (**B**) early-aborted colony associated with necrosis (EA+); (**C**) established colony without necrosis (EST−); (**D**) established colony associated with necrosis (EST+). A, appressorium; SSV, substomatal vesicle; HMC, haustorial mother cell; HR, hypersensitive response.

**Figure 3:**
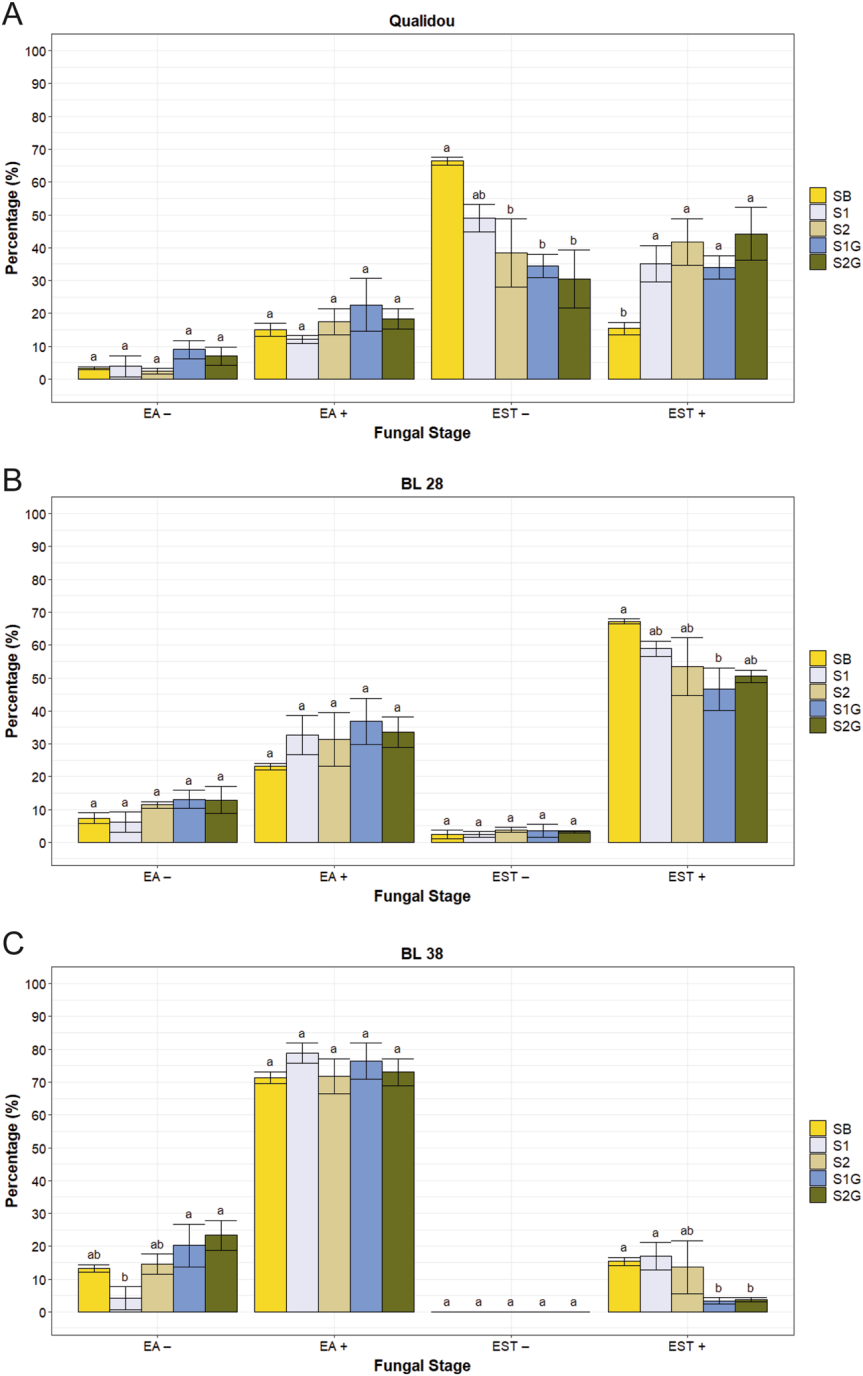
Microscopic fungal stages of *P. triticina* presented as mean percentages in three selected durum wheat accessions (**A**) Qualidou, (**B**) BL 28 and (**C**) BL 38 under baseline (SB) and climate change environments (S1, S2, S1G and S2G) at 5 dpi. Error bars represent the standard error calculated from three independent experiments with four replicates each. Data with the same letter within a fungal stage and accession are not significantly different (Duncan test, *p* < 0.05). EA−, early-aborted colonies without necrosis; EA + , early-aborted colonies with necrosis; EST−, established colonies without necrosis; EST + , established colonies with necrosis. SB: plants grown, inoculated, incubated, and maintained for evaluation under baseline weather conditions (24 °C and [CO_2_] around 420–450 ppm). S1 and S2: plants inoculated and incubated under baseline weather conditions, and then maintained for evaluation under far future weather conditions (S1, 30 °C and [CO_2_] around 420–450 ppm; S2, 30 °C and elevated [CO_2_] around 620–650 ppm). S1G and S2G: plants grown, inoculated, incubated, and maintained for evaluation under far future weather conditions (S1G, 30 °C and [CO_2_] around 420–450 ppm; S2G, 30 °C and elevated [CO_2_] around 620–650 ppm).

Different stages of fungal development were identified during microscopic evaluation of the *P. triticina* infection (EA−; EA+; EST−; EST+) (Fig. 2) and then analysed as percentages (Fig. 3 and Supplementary Table S3 and S4).

The susceptible accession Qualidou expressed the greatest differences amongst weather conditions (Fig. 3A). Thus, it could be observed that established colonies were the main fungal stage, and that presence (+) or absence (−) of necrotic cells in these colonies varied regarding sets. The percentage of EST− observed under environment B (SB, 66.38%) was significantly higher than those recorded for sets under far future weather conditions. In contrast, this accession showed the lowest EST+ percentage (15.36%) for the SB set, being this value statistically different compared with the higher ones exhibited under far future weather conditions. Despite Qualidou accession expressed a susceptible response against leaf rust, it also developed early-aborted colonies with (EA+) and without (EA−) the presence of necrosis, both non-significantly different amongst sets SB, S1, S2, S1G and S2G.

BL 28 accession also showed non-significant differences amongst fungal stages EA+ and EA− (Fig. 3B), although the presence of necrotic cells led to higher percentages of EA+ fungal stage for all sets. Fungal stage EST− presented the lowest values of the BL 28 accession, with an average score of 3.07% amongst sets. Lastly, EST+ (established colonies with necrosis) was the most abundant stage, showing the greatest differences between sets too. Thus, the highest EST+ percentage occurred for the SB set (67.20%), being the lowest percentage observed for S1G (46.66%).

The analysis of the resistant accession BL 38 (Fig. 3C) revealed the EA+ fungal stage as the most prominent one. However, non-statistically significant values amongst sets were shown. Oppositely, EST− fungal stage was absent. Significant differences amongst sets were just observed in the fungal stages EA− and EST+. BL 38 accession scored 4.20% in the S1 set for the EA− fungal stage, a value statistically different from values in S1G and S2G sets. Finally, the significantly lowest percentages for S1G and S2G sets were presented for the fungal stage EST+.

The colony size (CS) of established colonies (EST− and EST+) were also measured to study the effect of far future weather conditions in the microscopic colony development of leaf rust (Table 3). The susceptible accession Qualidou developed a compatible reaction to *P. triticina*, and exhibited diverse CS values amongst sets. In fact, plants exposed to environment B showed the highest CS value (SB set, 0.411 mm^2^), which was statistically different from CS values of the other sets. In addition, sets S1 and S2 showed statistically higher values than the ones in S1G and S2G. In contrast, BL 28 and BL 38 accessions showed moderately and highly resistant reactions to *P. triticina*, respectively, and did not generally develop statistically different values in CS amongst sets (Table 3).

**Table 3:** Colony Size (CS) of *P. triticina* established colonies (EST− and EST+) in three durum wheat selected accessions under baseline and climate change environments at 5 dpi.

Accession	Environmental set	Colony size (mm^2^)
Qualidou	SB	0.411 ± 0.012 a
	S1	0.351 ± 0.014 b
	S2	0.328 ± 0.018 b
	S1G	0.243 ± 0.022 c
	S2G	0.241 ± 0.018 c
BL 28	SB	0.191 ± 0.010 a
	S1	0.174 ± 0.005 ab
	S2	0.167 ± 0.023 ab
	S1G	0.151 ± 0.009 ab
	S2G	0.145 ± 0.009 b
BL 38	SB	0.112 ± 0.001 a
	S1	0.127 ± 0.001 a
	S2	0.122 ± 0.011 a
	S1G	0.113 ± 0.008 a
	S2G	0.112 ± 0.002 a

Values are mean ± standard error for four leaves evaluated for each accession and environmental set in three different experiments. Data with the same letter within an accession and column are not statistically different (Duncan test, *p* < 0.05). SB: plants grown, inoculated, incubated, and maintained for evaluation under baseline weather conditions (24 °C and [CO_2_] around 420–450 ppm). S1 and S2: plants inoculated and incubated under baseline weather conditions, and then maintained for evaluation under far future weather conditions (S1, 30 °C and [CO_2_] around 420–450 ppm; S2, 30 °C and elevated [CO_2_] around 620–650 ppm). S1G and S2G: plants grown, inoculated, incubated, and maintained for evaluation under far future weather conditions (S1G, 30 °C and [CO_2_] around 420–450 ppm; S2G, 30 °C and elevated [CO_2_] around 620–650 ppm).

## Discussion

Durum wheat cultivation is currently threatened by abiotic and biotic stresses. In fact, the increased risk of wheat leaf rust, coupled with the effects of increased temperature and [CO_2_] due to climate change, would lead to an uncertain scenario for durum wheat cultivation in the short and long term, especially in hotspots of climate change such as Mediterranean countries^17,26,56^. Although recent studies have evaluated the effects of elevated temperatures^41,63^ and elevated [CO_2_]^33,38^ in wheat-leaf rust interaction, not many have conducted diurnal fluctuating temperature cycles or even the combination of two abiotic factors. In this study, previous to the development of climate change studies, we first conducted an evaluation of leaf rust disease symptoms in a collection of 45 Spanish durum wheat breeding lines and commercial cultivars. Our results showed a general susceptible response (IT 7–9) amongst evaluated accessions, possibly due to the recent emergence of new virulent races in recent years not only in Spain^55^, but also in other Mediterranean countries such as France^64^. Particularly, the fact that the majority of breeding lines evaluated expressed a susceptible response against leaf rust is a quite concerning fact for the current Spanish breeding programs of durum wheat, which presented barely any sources of resistance against leaf rust^65^ in comparison with bread wheat^20,66^. However, some accessions, both breeding lines and commercial cultivars, expressed varied resistant responses, being valuable sources of resistance in future breeding programs^65,66^. Once all accessions were evaluated, we selected breeding line BL 38 (IT 1) as highly resistant, breeding line BL 28 (IT 4) as moderately resistant, and commercial cultivar Qualidou (IT 9) as susceptible for the development of climate change experiments.

### Disease development at elevated temperature (set S1)

Temperature is one of the most important factors influencing the life cycle of pathogens and their interactions with plants^40,67^. Our environment 1 was used to test the individual effect of increased maximum temperature in durum wheat-leaf rust interaction. To ensure the infection, plants of this assay were inoculated and incubated during 24 h under baseline weather conditions (plant set S1). In susceptible accession Qualidou, S1 plants expressed elevated IF values compared to plants which were grown, inoculated and incubated in environment B (SB plants). This suggests that elevated temperatures during plant growth resulted in physiological changes that favoured leaf rust penetration success and subsequent infection sites formation in Qualidou accession. Thus, higher temperatures increase the evapotranspiration rate^68^ and then, if the water status of the plant is correct, the stomatal aperture is induced, which favours fungal invasion of the host, as stomata are the main gateway for leaf rust to infect wheat^16^. This increased penetration success could be the reason for the slightly higher macroscopic values obtained in plants of BL 28 and BL 38 accessions at elevated temperatures.

Once established, fungal development was accelerated in Qualidou accession according to its shortened LP50 value compared to SB plants, a common effect of elevated temperatures in this pathogen^69^. This accelerated life cycle would increase the risk of higher sporulation, and subsequently the number of disease cycles, favouring the adaptation and appearance of new pathotypes^26,70,71^. However, Mean Pustule Size value in Qualidou accession was relevantly lower in S1 than in SB plants, confirmed microscopically by the lower CS value of established colonies and the elevated proportion of EST+ observed at 5 dpi. This resulted in increased chlorotic and necrotic areas (Total Disease Area value), which reduced photosynthesis and assimilates, leading leaf senescence and, subsequently, reducing yield potential in accession Qualidou. Therefore, considering the lower Pustule Development Rate value in comparison with SB plants, we might suggest this higher Total Disease Area value was due to host responses which restricted fungal development. Thus, considering also that temperature had little effect on pustule size^63^, the higher proportion of established colonies surrounded by necrotic cells (EST+) indicated the occurrence of some kind of host responses that restricted fungal development under elevated temperatures. Conversely, BL 28 (moderately resistant) and BL 38 (resistant) accessions did not show relevant differences for microscopic parameters compared to SB plants.

In this sense, prolonged exposure to abiotic stresses could lead to a priming or weakening of basal defence in plants prior to pathogen infection^31,48,67^. In fact, some durum wheat cultivars could prevent or minimise the detrimental effects of higher temperatures through basal or acquired (after acclimation) thermotolerance^50^, changing their transcriptome, proteome, metabolome or lipidome, and taking advantage against pathogens. Thus, prolonged elevated temperatures could reduce photosynthesis, generate ROSs, and trigger programmed cell death^6^. However, our observations detected this programmed cell death surrounding leaf rust colonies, indicating that supposed ROSs generation could act as a signalling molecule to mediate temperature stress responses^6^, such as induction of pathogen-associated defence genes^37,72^. Lastly, phytohormone crosstalk affected by elevated temperatures^3,47^, together with the generation of ROSs, could be a possible reason for elevated temperature-mediated cross-tolerance for subsequent leaf rust infections in Qualidou response^49^.

### Disease development at elevated temperature and [CO_2_] (set S2)

One of the most studied abiotic factors in plant diseases is elevated [CO_2_], but its effects vary from increasing^33,36,39^ to decreasing^35,38,73^ the incidence of different wheat diseases. In addition, few studies consider the effects of both elevated [CO_2_] and temperature together^34–37^, which would most closely resemble the effects of expected field climatic conditions in wheat-leaf rust interactions^26^. For that reason, plants of selected accessions were exposed to both elevated temperature and [CO_2_] (environment 2, 620–650 ppm) to assess their effects in the durum wheat-leaf rust interactions. To ensure infection, plants of this assay were inoculated and incubated during 24 h under baseline weather conditions (plant set S2).

Exposure of plants to elevated [CO_2_] for prolonged periods (days to weeks) reduces stomatal conductance and evapotranspiration, thereby increasing canopy temperature^74^. This situation forced S2 plants to develop a unique response during a subsequent leaf rust infection, which may reduce fungal penetration success through a partial stomatal closure^75,76^, confirmed by reduced IF values in Qualidou and BL 28 accessions in comparison with S1 plants. Despite this reduction, the IF parameter remained higher in the Qualidou accession (susceptible) compared to SB plants^33^. In addition, elevated [CO_2_] stimulates photosynthesis, especially in C_3_ plants as wheat^6^, increasing the production of sugar, starch and other carbohydrates^75,77^. On one hand, this additional carbohydrate accumulation in the leaf tissue could facilitate nutrient acquisition by the fungus^26,75,76^, as it was observed in the Qualidou accession with a reduction of the LP50 value under environment 2, in agreement with faster growth of other biotrophic fungus under elevated [CO_2_] conditions^73^. On the other hand, this carbohydrate production might act as an elicitor of defence responses such as enhancement of ROSs network and phytohormonal control^35,75^, as it was detected in Qualidou plants lower values of CS and EST−, coupled with higher values of EST + , and reduced values of Mean Pustule Size, Total Pustule Area, and Pustule Development Rate compared to SB plants.

Therefore, considering both elevated abiotic factors (temperature and [CO_2_]) induce changes in primary metabolism of plants through photosynthetic efficiency^34^, and elevated [CO_2_] may not increase wheat rusts incidence^38,71^, our results suggest that temperature was the main abiotic factor modulating the response of selected accessions against leaf rust infection. This may be due to plants prioritising their response to the most hampering abiotic stress^47^. Thus, it is feasible that elevated temperatures and [CO_2_] (environment 2), enhance those host physiological and molecular defence responses that are only expressed under elevated temperatures in the environment 1^35,40,75^. In fact, the susceptible accession Qualidou was the only one presenting some parameters with statistical differences in S2 plants compared to S1 plants. A lower Total Pustule Area value was shown, probably due to a lower IF value and, more likely, due to the aforementioned enhanced host defence responses that limited fungal development and subsequent pustule formation, and sporulation potential^26,70,71^. Interestingly, this lower value did not significantly reduce the Total Disease Area, supporting the fact that S2 Qualidou plants expressed some enhanced defence responses against leaf rust disease, although this would ultimately reduce host photosynthetic area and yield^14^.

### Inoculation, incubation and disease development at elevated temperature and [CO_2_] (sets S1G and S2G)

Leaf rust infection generally starts at night to ensure the correct disease establishment^78^. However, the even more frequent occurrence of heat waves coupled with changes in rainfall patterns caused by climate change^51,52^ would affect this crucial process to some extent, particularly in Mediterranean growing areas. For this reason, plants in sets S1G and S2G were grown, inoculated and incubated in environments 1 and 2, respectively, to assess the effect of abiotic factors on leaf rust disease establishment.

Interestingly, the three selected accessions showed a significant reduction in leaf rust symptoms in S1G and S2G plants compared to SB plants, especially for the macroscopic parameters IF, Total Pustule Area, Total Disease Area, and Pustule Development Rate. In fact, based on empirical studies^79–81^, the increased temperature reached during the inoculation process could affect the germination and penetration success of leaf rust spores, thus reducing disease symptoms. Therefore, it can be assumed that increments of maximum temperatures (30 °C) during the inoculation process could reduce spore germination by up to 50%^80^. This reduction could lead a delay in the disease establishment, explaining the relevantly longer LP50 values in S1G and S2G plants of the Qualidou accession, as opposed to those in S1 and S2 plants. In addition, no significant differences of EA− and EA + values were observed in comparison with SB plants in any accession, indicating a reduction of infection sites in S1G and S2G plants prior to the establishment of host–pathogen infection. Furthermore, considering that elevated temperatures (30–35 °C) did not negatively affect the fungus once it had entered the host^78^, our results suggest that the inoculation process for S1G and S2G plants could also slightly weaken the subsequent disease progression. This could be observed in the reduced proportion of EST− in the Qualidou accession and EST+ in BL 28 and BL 38 accessions, together with lower CS values in the three selected accessions, all values compared to SB plants. However, these data were statistically relevant mainly in Qualidou accession and only for EST+ in BL 38 accession.

Finally, S2G plants showed relevant differences in some macroscopic parameters compared to S1G plants, especially higher values of Total Disease Area for the three accessions, affecting more host photosynthetic area and reducing yield^14^. However, non-statistically significant differences in Total Pustule Area in Qualidou (susceptible) and BL 28 (moderately resistant) accessions were shown in S1G and S2G plants, suggesting that elevated [CO_2_] enhanced even more the host responses against leaf rust infection in S2G plants. This is particularly evident in the Qualidou accession, which also showed relevantly lower Mean Pustule Size and Pustule Development Rate values in comparison to S1G.

In conclusion, the most important fact in our study is that elevated maximum temperatures alone or in combination with elevated [CO_2_] did not suppress the general defence response in our studied accessions BL 28 (moderately resistant) and BL 38 (resistant), nor did it cause the loss of susceptibility in Qualidou plants during *P. triticina* infection. This suggests that the genetic resistance background of these accessions was not temperature-sensitive^71,82,83^ or the timing and intensity of abiotic stresses were not sufficient to affect it^26,32,84^. Therefore, variations in macro and microscopic components of resistance in plants exposed to environments 1 and 2 were due to abiotic factors affecting durum wheat-leaf rust interactions mainly through modifications in the host and/or pathogen biology and physiology^71^. In contrast, leaf rust disease was greatly reduced when plants were inoculated and incubated under environments 1 and 2 (S1G and S2G plants), mimicking possible future heat events and disturbed rainfall patterns, suggesting that climate change would affect key stages of *P. triticina* and thus subsequent disease incidence in Mediterranean regions.

### Supplementary Information

Supplementary Tables.

Supplementary Figure S1.

## Data Availability

All data generated or analysed during this study are included in this published article and its Supplementary Materials. Correspondence and requests for materials should be addressed to A.P.L. The study complies with local and national regulations.

## References

[1] FAOSTAT. *Food and Agriculture Organization of the United Nations Statistical Database*. Available online: https://www.fao.org/faostat/es/. Accessed 01 March 2023.

[2] World Bank. *World Development Indicators: Data Bank*. Available online: https://databank.worldbank.org/reports.aspx?source=World-Development-Indicators. Accessed 01 March 2023.

[3] Abhinandan K (2018). Abiotic stress signaling in wheat—An inclusive overview of hormonal interactions during abiotic stress responses in wheat. Front. Plant Sci..

[4] Hossain A (2021). Consequences and mitigation strategies of abiotic stresses in wheat (*Triticum*
*aestivum* L.) under the changing climate. Agronomy.

[5] Pachauri RK, Pachauri RK, Meyer L (2014). Climate change 2014: Synthesis report. Contribution of Working Groups I, II and III to the Fifth Assessment Report of the Intergovernmental Panel on Climate Change.

[6] Chaudhry S, Sidhu GPS (2022). Climate change regulated abiotic stress mechanisms in plants: A comprehensive review. Plant Cell Rep..

[7] Jacob D (2014). EURO-CORDEX: New high-resolution climate change projections for European impact research. Reg. Environ. Change.

[8] Oerke EC (2006). Crop losses to pests. J. Agric. Sci..

[9] Savary S (2019). The global burden of pathogens and pests on major food crops. Nat. Ecol. Evol..

[10] Strange RN, Scott PR (2005). Plant disease: A threat to global food security. Annu. Rev. Phytopathol..

[11] Figueroa M, Hammond-Kosack KE, Solomon PS (2018). A review of wheat diseases—A field perspective. Mol. Plant Pathol..

[12] Sharma I, Sharma I (2012). Disease in wheat crops—An introduction. Disease Resistance in Wheat.

[13] Singh RP (2016). Disease impact on wheat yield potential and prospects of genetic control. Annu. Rev. Phytopathol..

[14] Chai Y, Pardey PG, Hurley TM, Senay SD, Beddow JM (2020). A probabilistic bio-economic assessment of the global consequences of wheat leaf rust. Phytopathology.

[15] Huerta-Espino J (2011). Global status of wheat leaf rust caused by *Puccinia*
*triticina*. Euphytica.

[16] Bolton MD, Kolmer JA, Garvin DF (2008). Wheat leaf rust caused by *Puccinia*
*triticina*. Mol. Plant Pathol..

[17] Pérez-Méndez N (2022). Plant breeding and management strategies to minimize the impact of water scarcity and biotic stress in cereal crops under mediterranean conditions. Agronomy.

[18] McIntosh, R. *et al*. *Catalogue of Gene Symbols for Wheat 2020—Supplement 2017*. Available online: https://wheat.pw.usda.gov/GG3/Wheat_Gene_Catalog_Documents. Accessed 01 March 2023.

[19] Martínez-Moreno F, Giraldo P, Nieto C, Ruiz M (2022). Resistance to leaf and yellow rust in a collection of Spanish bread wheat landraces and association with ecogeographical variables. Agronomy.

[20] Soleiman NH (2014). Evaluation of macroscopic and microscopic components of partial resistance to leaf rust in durum wheat. J. Phytopathol..

[21] Cabrera A, Porras R, Palomino C, Sillero JC (2023). Introgression of seedling plant resistance to leaf rust from *Agropyron*
*cristatum* into wheat by induced homoeologous recombination. Agronomy.

[22] Gallego-Sánchez LM, Canales FJ, Montilla-Bascón G, Prats E (2020). Rust: A robust, user-friendly script tool for rapid measurement of rust disease on cereal leaves. Plants.

[23] Porras R, Miguel-Rojas C, Pérez-de-Luque A, Sillero JC (2022). Macro- and microscopic characterization of components of resistance against *Puccinia*
*striiformis* f. sp. tritici in a collection of Spanish bread wheat cultivars. Agronomy.

[24] Wang X (2013). Comparative microscopic and molecular analysis of Thatcher near-isogenic lines with wheat leaf rust resistance genes *Lr2a*, *Lr3*, *LrB* or *Lr9* upon challenge with different *Puccinia*
*triticina* races. Plant Pathol..

[25] Wesp-Guterres C, Martinelli JA, Graichen FAS, Chaves MS (2013). Histopathology of durable adult plant resistance to leaf rust in the Brazilian wheat variety Toropi. Eur. J. Plant Pathol..

[26] Velásquez AC, Castroverde CDM, He SY (2018). Plant–pathogen warfare under changing climate conditions. Curr. Biol..

[27] Juroszek P, von Tiedemann A (2013). Climate change and potential future risks through wheat diseases: A review. Eur. J. Plant Pathol..

[28] Juroszek P, Von Tiedemann A (2015). Linking plant disease models to climate change scenarios to project future risks of crop diseases: A review. J. Plant Dis. Prot..

[29] Miedaner T, Juroszek P (2021). Climate change will influence disease resistance breeding in wheat in Northwestern Europe. Theor. Appl. Genet..

[30] Gouache D (2013). Modelling climate change impact on *Septoria tritici* blotch (STB) in France: Accounting for climate model and disease model uncertainty. Agric. For. Meteorol..

[31] Ramegowda V, Senthil-Kumar M (2015). The interactive effects of simultaneous biotic and abiotic stresses on plants: Mechanistic understanding from drought and pathogen combination. J. Plant Physiol..

[32] Suzuki N, Rivero RM, Shulaev V, Blumwald E, Mittler R (2014). Abiotic and biotic stress combinations. New Phytol..

[33] Bencze S, Vida G, Balla K, Varga-László E, Veisz O (2013). Response of wheat fungal diseases to elevated atmospheric CO_2_ level. Cereal Res. Commun..

[34] Hay WT, McCormick SP, Vaughan MM (2021). Effects of atmospheric CO_2_ and temperature on wheat and corn susceptibility to *Fusarium*
*graminearum* and deoxynivalenol contamination. Plants.

[35] Matić S, Cucu MA, Garibaldi A, Gullino ML (2018). Combined effect of CO_2_ and temperature on wheat powdery mildew development. Plant Pathol. J..

[36] Melloy P, Aitken E, Luck J, Chakraborty S, Obanor F (2014). The influence of increasing temperature and CO_2_ on Fusarium crown rot susceptibility of wheat genotypes at key growth stages. Eur. J. Plant Pathol..

[37] Mikkelsen BL, Jørgensen RB, Lyngkjær MF (2015). Complex interplay of future climate levels of CO_2_, ozone and temperature on susceptibility to fungal diseases in barley. Plant Pathol..

[38] Tiedemann AV, Firsching KH (2000). Interactive effects of elevated ozone and carbon dioxide on growth and yield of leaf rust-infected versus non-infected wheat. Environ. Pollut..

[39] Váry Z, Mullins E, Mcelwain JC, Doohan FM (2015). The severity of wheat diseases increases when plants and pathogens are acclimatized to elevated carbon dioxide. Glob. Change Biol..

[40] Cheng C (2013). Plant immune response to pathogens differs with changing temperatures. Nat. Commun..

[41] Kolmer JA (1996). Genetics of resistance to wheat leaf rust. Annu. Rev. Phytopathol..

[42] Qayoum A, Line RF (1985). High-temperature, adult-plant resistance to stripe rust of wheat. Phytopathology.

[43] Gousseau HDM, Deverall BJ, McIntosh RA (1985). Temperature-sensitivity of the expression of resistance to *Puccinia*
*graminis* conferred by the *Sr15*, *Sr9b* and *Sr14* genes in wheat. Physiol. Plant Pathol..

[44] El-Orabey W, Shaheen D, Mabrouk O, Elkot A, Esmail S (2020). Effect of temperature on monogenic lines of wheat leaf rust caused by *Puccinia*
*triticina*. Egypt. J. Agron..

[45] Bryant RRM (2014). A change in temperature modulates defence to yellow (stripe) rust in wheat line UC1041 independently of resistance gene *Yr36*. BMC Plant Biol..

[46] Zhang W (2017). Identification and characterization of *Sr13*, a tetraploid wheat gene that confers resistance to the Ug99 stem rust race group. Proc. Natl. Acad. Sci. U.S.A..

[47] Atkinson NJ, Jain R, Urwin PE, Mahalingam R (2015). The response of plants to simultaneous biotic and abiotic stress. Combined Stresses in Plants.

[48] Bostock RM, Pye MF, Roubtsova TV (2014). Predisposition in plant disease: Exploiting the nexus in abiotic and biotic stress perception and response. Annu. Rev. Phytopathol..

[49] Ben Rejeb I, Pastor V, Mauch-Mani B (2014). Plant responses to simultaneous biotic and abiotic stress: molecular mechanisms. Plants.

[50] Rampino P (2009). Acquisition of thermotolerance and HSP gene expression in durum wheat (*Triticum*
*durum* Desf.) cultivars. Environ. Exp. Bot..

[51] Diffenbaugh NS, Giorgi F (2012). Climate change hotspots in the CMIP5 global climate model ensemble. Clim. Change.

[52] Trnka M (2014). Adverse weather conditions for European wheat production will become more frequent with climate change. Nat. Clim. Change.

[53] Ceglar A, Toreti A, Zampieri M, Royo C (2021). Global loss of climatically suitable areas for durum wheat growth in the future. Environ. Res. Lett..

[54] Xynias IN (2020). Durum wheat breeding in the Mediterranean region: Current status and future prospects. Agronomy.

[55] Soleiman NH (2016). Emergence of a new race of leaf rust with combined virulence to *Lr14a* and *Lr72* genes on durum wheat. Span. J. Agric. Res..

[56] Lorite IJ (2023). Analyzing the impact of extreme heat events and drought on wheat yield and protein concentration, and adaptation strategies using long-term cultivar trials under semi-arid conditions. Agric. For. Meteorol..

[57] McNeal FH, Konzak CF, Smith EP, Tate WS, Russell TS (1971). A uniform system for recording and processing cereal research data. US Agric. Res. Serv..

[58] Porras R, Miguel-Rojas C, Lorite IJ, Pérez-de-Luque A, Sillero JC (2023). Characterization of durum wheat resistance against septoria tritici blotch under climate change conditions of increasing temperature and CO_2_ concentration. Agronomy.

[59] Sørensen CK, Thach T, Hovmøller MS, Periyannan S (2017). Assessment of aggressiveness of *Puccinia**striiformis* on wheat. Wheat Rust Diseases. Methods in Molecular Biology.

[60] Schindelin J (2012). Fiji: An open-source platform for biological-image analysis. Nat. Methods.

[61] Rubiales D, Niks RE (1995). Characterization of *Lr34*, a major gene conferring nonhypersensitive resistance to wheat leaf rust. Plant Dis..

[62] R Core Team. *R: A language and environment for statistical computing*. Available online: https://www.r-project.org/ (R Found. Stat. Comput. Vienna, 2022).

[63] Singh R, Huerta-Espino J (2003). Effect of leaf rust resistance gene *Lr34* on components of slow rusting at seven growth stages in wheat. Euphytica.

[64] Goyeau H (2012). Low diversity and fast evolution in the population of *Puccinia*
*triticina* causing durum wheat leaf rust in France from 1999 to 2009, as revealed by an adapted differential set. Plant Pathol..

[65] Martínez-Moreno F, Giraldo P, Cátedra MDM, Ruiz M (2021). Evaluation of leaf rust resistance in the Spanish core collection of tetraploid wheat landraces and association with ecogeographical variables. Agriculture.

[66] Soleiman NH (2014). Resistance to leaf rust in a set of durum wheat cultivars and landraces in Spain. J. Plant Pathol..

[67] Pandey P, Irulappan V, Bagavathiannan MV, Senthil-Kumar M (2017). Impact of combined abiotic and biotic stresses on plant growth and avenues for crop improvement by exploiting physio-morphological traits. Front. Plant Sci..

[68] Swelam A, Jomaa I, Shapland T, Snyder RL, Moratiel R, Fernández JE, Ferreira MI (2011). Evapotranspiration response to climate change. XXVIII International Horticultural Congress on Science and Horticulture for People (IHC2010): International Symposium on CLIMWATER 2010: Horticultural Use of Water in a Changing Climate.

[69] Wójtowicz A, Wójtowicz M, Sigvald R, Pasternak M (2017). Predicting the effects of climate change on the latency period of wheat leaf rust in western Poland. Acta Agric. Scand. Sect. B Soil Plant Sci..

[70] Chakraborty S (2013). Migrate or evolve: Options for plant pathogens under climate change. Glob. Change Biol..

[71] Chakraborty S, Luck J, Hollaway G, Fitzgerald G, White N (2011). Rust-proofing wheat for a changing climate. Euphytica.

[72] Petrov V, Hille J, Mueller-Roeber B, Gechev TS (2015). ROS-mediated abiotic stress-induced programmed cell death in plants. Front. Plant Sci..

[73] Hibberd JM, Whitbread R, Farrar JF (1996). Effect of elevated concentrations of CO_2_ on infection of barley by *Erysiphe*
*graminis*. Physiol. Mol. Plant Pathol..

[74] Kimball BA, Bernacchi CJ, Nörsberger J (2006). Evapotranspiration, canopy temperature, and plant water relations. Managed Ecosystems and CO2. Ecological Studies.

[75] Eastburn DM, McElrone AJ, Bilgin DD (2011). Influence of atmospheric and climatic change on plant-pathogen interactions. Plant Pathol..

[76] Manning WJ, Tiedemann A (1995). Climate change: Potential effects of increased atmospheric Carbon dioxide (CO_2_), ozone (O_3_), and ultraviolet-B (UV-B) radiation on plant diseases. Environ. Pollut..

[77] Loladze I (2014). Hidden shift of the ionome of plants exposed to elevated CO_2_ depletes minerals at the base of human nutrition. Elife.

[78] Singh RP, Huerta-Espino J, Roelfs AP, Curtis BC, Rajaram S, Gómez Macpherson H (2002). The wheat rusts. Bread Wheat. Improvement and Production.

[79] de Vallavieille-Pope C, Huber L, Leconte M, Goyeau H (1995). Comparative effects of temperature and interrupted wet periods on germination, penetration, and infection of *Puccinia*
*recondita* f. sp. tritici and *P.*
*striiformis* on wheat seedlings. Phytopathology.

[80] Kramer CL, Eversmeyer MG (1992). Effect of temperature on germination and germ-tube development of *Puccinia*
*recondita* and *P.*
*graminis* urediniospores. Mycol. Res..

[81] Wiese MV (1979). Environmental effects on inoculum quality of dormant rust uredospores. Phytopathology.

[82] Kaul K, Shaner G (1989). Effect of temperature on adult-plant resistance to leaf rust in wheat. Phytopathology.

[83] Statler GD, Christianson T (1993). Temperature studies with wheat leaf rust. Can. J. Plant Pathol..

[84] Kane K (2013). Long-term growth under elevated CO_2_ suppresses biotic stress genes in non-acclimated, but not cold-acclimated winter wheat. Plant Cell Physiol..

