# Supplementary material for: Characterization of durum wheat resistance against leaf rust under climate change conditions of increasing temperature and [CO2]
Source: Sci Rep. 2023 Dec 12;13:22001. doi: 10.1038/s41598-023-49118-w (PMC10713590; doi:10.1038/s41598-023-49118-w)
Supplement: Supplementary file 1 — Supplementary Tables. [file 41598_2023_49118_MOESM1_ESM.docx]

**Table S1.** Commercial Cultivars and breeding lines used in this work and their source of origin.

| **Breeding lines** | |  | **Commercial cultivars** | |
| --- | --- | --- | --- | --- |
| **Accession** | **Breeding**  **program**  **origin** |  | **Accession** | **Company** |
| BL 27 | IFAPA^[1]^ |  | Amilcar | Semillas Guadalsem S.L. |
| BL 28 | CIMMYT^[2]^ |  | Athoris | Limagrain Ibérica S.A. |
| BL 30 | CIMMYT |  | Avispa | Limagrain Ibérica S.A. |
| BL 31 | IFAPA |  | Don Ortega | Agrovegetal S.A. |
| BL 32 | IFAPA |  | Don Ricardo | Agrovegetal S.A. |
| BL 33 | IFAPA |  | Egeo | MAS Seeds |
| BL 34 | CIMMYT |  | Fuego | Semillas Guadalsem S.L. |
| BL 36 | CIMMYT |  | Kiko Nick | Limagrain Ibérica S.A. |
| BL 38 | CIMMYT |  | LG Acropolis | Limagrain Ibérica S.A. |
| BL 39 | CIMMYT |  | LG Hipnosis | Limagrain Ibérica S.A. |
| BL 40 | CIMMYT |  | LG Origen | Limagrain Ibérica S.A. |
| BL 41 | CIMMYT |  | Qualidou | Florimond Desprez Ibérica S.A. |
| BL 43 | CIMMYT |  | RGT Beticur | RAGT Ibérica S.L.U. |
| BL 44 | IFAPA |  | RGT Fernandur | RAGT Ibérica S.L.U. |
| BL 45 | IFAPA |  | RGT Leondur | RAGT Ibérica S.L.U. |
| BL 46 | CIMMYT |  | RGT Rumbadur | RAGT Ibérica S.L.U. |
| BL 47 | IFAPA |  | RGT Voilur | RAGT Ibérica S.L.U. |
| BL 48 | IFAPA |  | RGT Xiriur | RAGT Ibérica S.L.U. |
| BL 49 | CIMMYT |  | Salgado | AGRUSA |
| BL 50 | CIMMYT |  | Sculptur | RAGT Ibérica S.L.U. |
| BL 51 | CIMMYT |  | Simeto | PROSEME S.R.L. |
| BL 52 | IFAPA |  | Sy Leonardo | Syngenta S.A. |
|  |  |  | Teodorico | MAS Seeds |

^[1]^ Instituto Andaluz de Investigación y Formación Agraria, Pesquera, Alimentaria y de la Producción Ecológica. Centro Alameda del Obispo, Córdoba (Spain)

^[2]^ Centro Internacional de Mejoramiento de Maíz y Trigo, El Batán, Texcoco, México.

**Table S2.** Infection types (IT) at third-leaf stage on 37 near-isogenic Thatcher lines with known *R*-genes inoculated with the SanEs18/5 *P. triticina* isolate ^1^.

| **Pedigree / Line** | **Resistance gene** | **Response to leaf rust** | **Infection Type (IT)** |
| --- | --- | --- | --- |
| Thatcher | *–* | Susceptible | 9 |
| TcLr1 | *Lr1* | Susceptible | 9 |
| TcLr2a | *Lr2a* | Resistant | 2 |
| TcLr2b | *Lr2b* | Resistant | 6 |
| TcLr2c | *Lr2c* | Susceptible | 9 |
| TcLr3 | *Lr3* | Susceptible | 9 |
| TcLr3bg | *Lr3bg* | Susceptible | 9 |
| TcLr3ka | *Lr3ka* | Susceptible | 9 |
| TcLr9 | *Lr9* | Resistant | 1 |
| TcLr10 | *Lr10* | Susceptible | 9 |
| TcLr11 | *Lr11* | Susceptible | 9 |
| TcLr12 | *Lr12* | Susceptible | 9 |
| TcLr13 | *Lr13* | Resistant | 6 |
| TcLr14a | *Lr14a* | Susceptible | 9 |
| TcLr14b | *Lr14b* | Susceptible | 9 |
| TcLr15 | *Lr15* | Resistant | 2 |
| TcLr16 | *Lr16* | Resistant | 6 |
| TcLr17 | *Lr17* | Resistant | 6 |
| TcLr18 | *Lr18* | Susceptible | 9 |
| TcLr19 | *Lr19* | Resistant | 1 |
| TcLr20 | *Lr20* | Susceptible | 9 |
| TcLr21 | *Lr21* | Resistant | 6 |
| TcLr22a | *Lr22a* | Susceptible | 9 |
| TcLr23 | *Lr23* | Susceptible | 9 |
| TcLr24 | *Lr24* | Resistant | 1 |
| TcLr25 | *Lr25* | Resistant | 1 |
| TcLr26 | *Lr26* | Resistant | 2 |
| TcLr28 | *Lr28* | Resistant | 1 |
| TcLr30 | *Lr30* | Susceptible | 9 |
| TcLr32 | *Lr32* | Resistant | 6 |
| TcLr33 | *Lr33* | Susceptible | 9 |
| TcLr34 | *Lr34* | Susceptible | 9 |
| TcLr35 | *Lr35* | Susceptible | 9 |
| TcLr36 | *Lr36* | Resistant | 2 |
| TcLr37 | *Lr37* | Susceptible | 9 |
| TcLr45 | *Lr45* | Susceptible | 9 |
| TcLrB | *LrB* | Susceptible | 9 |
| TcLrW | *LrW* | Resistant | 1 |

^1^ Values evaluated for four plants for each line.

**Table S3.** Microscopic fungal stages of *P. triticina* infection in three selected durum wheat accessions under baseline and climate change environments at 5 dpi ^1^.

| **Accession** | **Environmental Set** | **Experiment** | **EA– (%)** | **EA+ (%)** | **EST– (%)** | **EST+ (%)** |
| --- | --- | --- | --- | --- | --- | --- |
| Qualidou | SB | 1 | 3.37 | 15.17 | 67.98 | 13.48 |
|  |  | 2 | 4.12 | 18.24 | 64.12 | 13.53 |
|  |  | 3 | 2.31 | 11.56 | 67.05 | 19.08 |
|  | S1 | 1 | 0.57 | 14.37 | 40.80 | 44.25 |
|  |  | 2 | 0.56 | 11.30 | 51.98 | 36.16 |
|  |  | 3 | 10.40 | 10.40 | 54.34 | 24.86 |
|  | S2 | 1 | 0.62 | 23.60 | 25.47 | 50.31 |
|  |  | 2 | 2.96 | 18.93 | 30.77 | 47.34 |
|  |  | 3 | 3.47 | 9.83 | 58.96 | 27.75 |
|  | S1G | 1 | 6.71 | 38.26 | 27.52 | 27.52 |
|  |  | 2 | 5.59 | 18.01 | 36.65 | 39.75 |
|  |  | 3 | 14.56 | 11.39 | 39.24 | 34.81 |
|  | S2G | 1 | 5.49 | 23.17 | 16.46 | 54.88 |
|  |  | 2 | 2.94 | 19.41 | 28.24 | 49.41 |
|  |  | 3 | 12.43 | 12.43 | 46.75 | 28.40 |
| BL 28 | SB | 1 | 4.17 | 25.00 | 2.98 | 67.86 |
|  |  | 2 | 8.59 | 21.47 | 4.29 | 65.64 |
|  |  | 3 | 9.20 | 22.70 | 0.00 | 68.10 |
|  | S1 | 1 | 0.00 | 43.67 | 1.90 | 54.43 |
|  |  | 2 | 8.43 | 30.72 | 1.20 | 59.64 |
|  |  | 3 | 9.94 | 23.39 | 4.09 | 62.57 |
|  | S2 | 1 | 13.19 | 38.19 | 4.86 | 43.75 |
|  |  | 2 | 11.11 | 40.74 | 2.47 | 45.68 |
|  |  | 3 | 9.88 | 15.12 | 4.07 | 70.93 |
|  | S1G | 1 | 17.69 | 47.62 | 0.00 | 34.69 |
|  |  | 2 | 8.44 | 38.96 | 3.90 | 48.70 |
|  |  | 3 | 13.16 | 23.68 | 6.58 | 56.58 |
|  | S2G | 1 | 6.82 | 36.36 | 3.41 | 53.41 |
|  |  | 2 | 11.04 | 39.61 | 2.60 | 46.75 |
|  |  | 3 | 20.73 | 24.39 | 3.66 | 51.22 |
| BL 38 | SB | 1 | 11.32 | 74.84 | 0.00 | 13.84 |
|  |  | 2 | 13.38 | 68.79 | 0.00 | 17.83 |
|  |  | 3 | 15.17 | 70.34 | 0.00 | 14.48 |
|  | S1 | 1 | 0.70 | 84.51 | 0.00 | 14.79 |
|  |  | 2 | 11.19 | 77.62 | 0.00 | 11.19 |
|  |  | 3 | 0.69 | 74.31 | 0.00 | 25.00 |
|  | S2 | 1 | 16.91 | 80.15 | 0.00 | 2.94 |
|  |  | 2 | 18.18 | 73.43 | 0.00 | 8.39 |
|  |  | 3 | 8.59 | 61.96 | 0.00 | 29.45 |
|  | S1G | 1 | 31.85 | 66.67 | 0.00 | 1.48 |
|  |  | 2 | 9.22 | 85.82 | 0.00 | 4.96 |
|  |  | 3 | 19.71 | 76.64 | 0.00 | 3.65 |
|  | S2G | 1 | 19.08 | 77.63 | 0.00 | 3.29 |
|  |  | 2 | 18.44 | 76.60 | 0.00 | 4.96 |
|  |  | 3 | 32.41 | 64.81 | 0.00 | 2.78 |

^1^ Values evaluated for four leaves for each experiment, environmental set and accession. EA–, early-aborted colonies without necrosis; EA+, early-aborted colonies with necrosis; EST–, established colonies without necrosis; EST+, established colonies with necrosis. SB: plants grown, inoculated, incubated, and maintained for evaluation under baseline weather conditions (24 °C and [CO_2_] around 420–450 ppm). S1 and S2: plants inoculated and incubated under baseline weather conditions, and then maintained for evaluation under far future weather conditions (S1, 30 °C and [CO_2_] around 420–450 ppm; S2, 30 °C and elevated [CO_2_] around 620–650 ppm). S1G and S2G: plants grown, inoculated, incubated, and maintained for evaluation under far future weather conditions (S1G, 30 °C and [CO_2_] around 420–450 ppm; S2G, 30 °C and elevated [CO_2_] around 620–650 ppm).

**Table S4.** Microscopic fungal stages of *P. triticina* infection in three selected durum wheat accessions under baseline and climate change environments at 5 dpi ^1^.

| **Accession** | **Environmental Set** | **EA– (%)** | **EA+ (%)** | **EST– (%)** | **EST+ (%)** |
| --- | --- | --- | --- | --- | --- |
| Qualidou | SB | 3.27 ± 0.52 a | 14.99 ± 1.93 a | 66.38 ± 1.16 a | 15.36 ± 1.86 b |
|  | S1 | 3.85 ± 3.28 a | 12.02 ± 1.20 a | 49.04 ± 4.17 ab | 35.09 ± 5.63 a |
|  | S2 | 2.35 ± 0.88 a | 17.45 ± 4.05 a | 38.40 ± 10.39 b | 41.80 ± 7.08 a |
|  | S1G | 8.95 ± 2.82 a | 22.55 ± 8.08 a | 34.47 ± 3.56 b | 34.03 ± 3.55 a |
|  | S2G | 6.95 ± 2.83 a | 18.34 ± 3.15 a | 30.48 ± 8.81 b | 44.23 ± 8.07 a |
| BL 28 | SB | 7.32 ± 1.59 a | 23.06 ± 1.03 a | 2.42 ± 1.27 a | 67.20 ± 0.78 a |
|  | S1 | 6.13 ± 3.09 a | 32.60 ± 5.93 a | 2.40 ± 0.87 a | 58.88 ± 2.38 ab |
|  | S2 | 11.40 ± 0.97 a | 31.35 ± 8.15 a | 3.80 ± 0.70 a | 53.45 ± 8.76 ab |
|  | S1G | 13.10 ± 2.67 a | 36.75 ± 7.00 a | 3.49 ± 1.91 a | 46.66 ± 6.40 b |
|  | S2G | 12.86 ± 4.12 a | 33.45 ± 4.63 a | 3.22 ± 0.32 a | 50.46 ± 1.96 ab |
| BL 38 | SB | 13.29 ± 1.11 ab | 71.33 ± 1.81 a | 0.00 ± 0.00 a | 15.38 ± 1.24 a |
|  | S1 | 4.20 ± 3.50 b | 78.81 ± 3.00 a | 0.00 ± 0.00 a | 16.99 ± 4.14 a |
|  | S2 | 14.56 ± 3.01 ab | 71.85 ± 5.31 a | 0.00 ± 0.00 a | 13.59 ± 8.08 ab |
|  | S1G | 20.26 ± 6.54 a | 76.37 ± 5.53 a | 0.00 ± 0.00 a | 3.37 ± 1.02 b |
|  | S2G | 23.31 ± 4.55 a | 73.01 ± 4.11 a | 0.00 ± 0.00 a | 3.68 ± 0.66 b |

^1^ Values are mean ± standard error for four leaves evaluated for each environmental set in three different experiments. Data with the same letter within an accession and column are not statistically different (Duncan test, *p* < 0.05). EA–, early-aborted colonies without necrosis; EA+, early-aborted colonies with necrosis; EST–, established colonies without necrosis; EST+, established colonies with necrosis. SB: plants grown, inoculated, incubated, and maintained for evaluation under baseline weather conditions (24 °C and [CO_2_] around 420–450 ppm). S1 and S2: plants inoculated and incubated under baseline weather conditions, and then maintained for evaluation under far future weather conditions (S1, 30 °C and [CO_2_] around 420–450 ppm; S2, 30 °C and elevated [CO_2_] around 620–650 ppm). S1G and S2G: plants grown, inoculated, incubated, and maintained for evaluation under far future weather conditions (S1G, 30 °C and [CO_2_] around 420–450 ppm; S2G, 30 °C and elevated [CO_2_] around 620–650 ppm).
