# Supplementary material for: Characterization of durum wheat resistance against leaf rust under climate change conditions of increasing temperature and [CO2]
Source: Sci Rep. 2023 Dec 12;13:22001. doi: 10.1038/s41598-023-49118-w (PMC10713590; doi:10.1038/s41598-023-49118-w)
Supplement: Supplementary file 2 — Supplementary Figure S1. [file 41598_2023_49118_MOESM2_ESM.pdf]

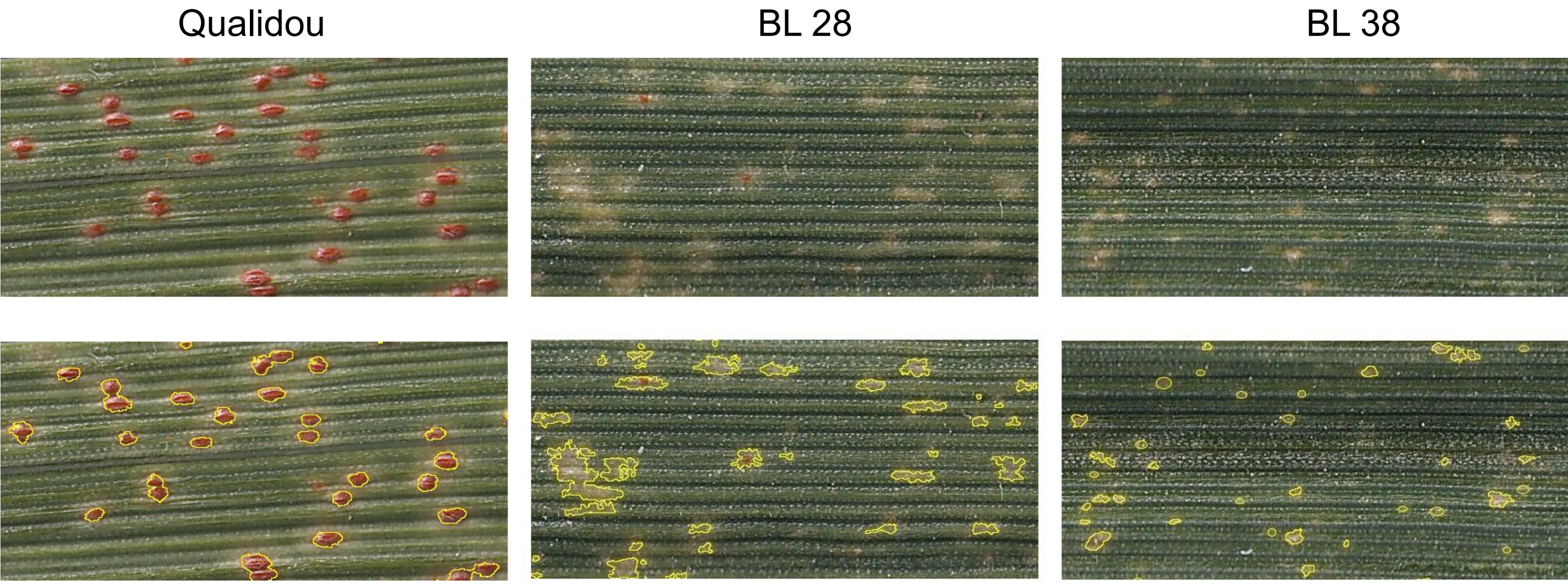

Figure S1. Output example of *P. triticina* image analysis in the studied durum wheat accessions Qualidou, BL 28 and, BL 38. Upper panels show original leaves. Lower panes show the analysed leaves in which pustules and chlorotic and necrotic lesions caused by *P. triticina* were selected in yellow colour.
